# HIGH-INTENSITY WALKING EXERCISE REDUCES FRAILTY AMONG PREFRAIL AND FRAIL OLDER ADULTS

**DOI:** 10.1093/geroni/igad104.1655

**Published:** 2023-12-21

**Authors:** Margaret Danilovich, Daniel Corcos, Ying Cheung, David Conroy, Lauren Balmert Bonner

**Affiliations:** CJE SeniorLife, Chicago, Illinois, United States; Northwestern University, Chicago, Illinois, United States; Northwestern University, Chicago, Illinois, United States; Penn State University, State College, Pennsylvania, United States; Northwestern University, Chicago, Illinois, United States

## Abstract

Exercise is the most recommended therapeutic intervention for frailty, yet the precise exercise prescription remains elusive. The purpose of this study was to use a cluster randomized controlled trial to evaluate the impact of exercise intensity on frailty in older adults. We tested a high (HIW) vs. low (LIW) intensity walking intervention among n=165 pre-frail and frail residents from n=12 independent living communities. The 3x/week, 4 month supervised intervention targeted >70% heart rate reserve for HIW participants and < 50% heart rate reserve for LIW participants. The primary outcome was frailty category improvement as assessed by the SHARE-FI. We measured usual and fast gait speed, 6-minute walk test, Timed Up and Go, SPPB, Berg Balance, Falls Efficacy, and PROMIS Global Health. Participants were on average 79 years of age, 79% female, and 75% white. We analyzed outcomes based on a modified intention-to-treat principle using a linear or generalized linear mixed model with fixed arm, baseline outcome measure score and random cluster effects. We found increased odds of improving at least one SHARE-FI frailty category in the HIW group (OR=2.91 [reference group=LIW], 95% CI 1.19,7.13, p=0.02, N=103). Among secondary outcomes, there was a significant difference in change in usual gait speed (mean difference = 0.078, 95% CI = 0.026, 0.131, Hochberg adjusted P-value = 0.0357), as well as 6-minute walk test (mean difference = 160.72, 95% CI = 69.2, 252.2, Hochberg adjusted P-value = 0.0075). Results suggest that older adults should engage in more intensive/higher heart rate cardiovascular exercise to reduce frailty.

